# Supplementary material for: DNA duplication-mediated activation of a two-component regulatory system serves as a bet-hedging strategy for Burkholderia thailandensis
Source: bioRxiv. 2024 Dec 9:2024.12.09.627470. Preprint. [Version 1] doi: 10.1101/2024.12.09.627470 (PMC11661271; doi:10.1101/2024.12.09.627470)
Supplement: Supplement 1 [file NIHPP2024.12.09.627470v1-supplement-1.pdf]

bioRxiv preprint doi: https://doi.org/10.1101/2024.12.09.627470; this version posted December 9, 2024. The copyright holder for this preprint (which was not certified by peer review) is the author/funder, who has granted bioRxiv a license to display the preprint in perpetuity. It is made available under aCC-BY 4.0 International license.

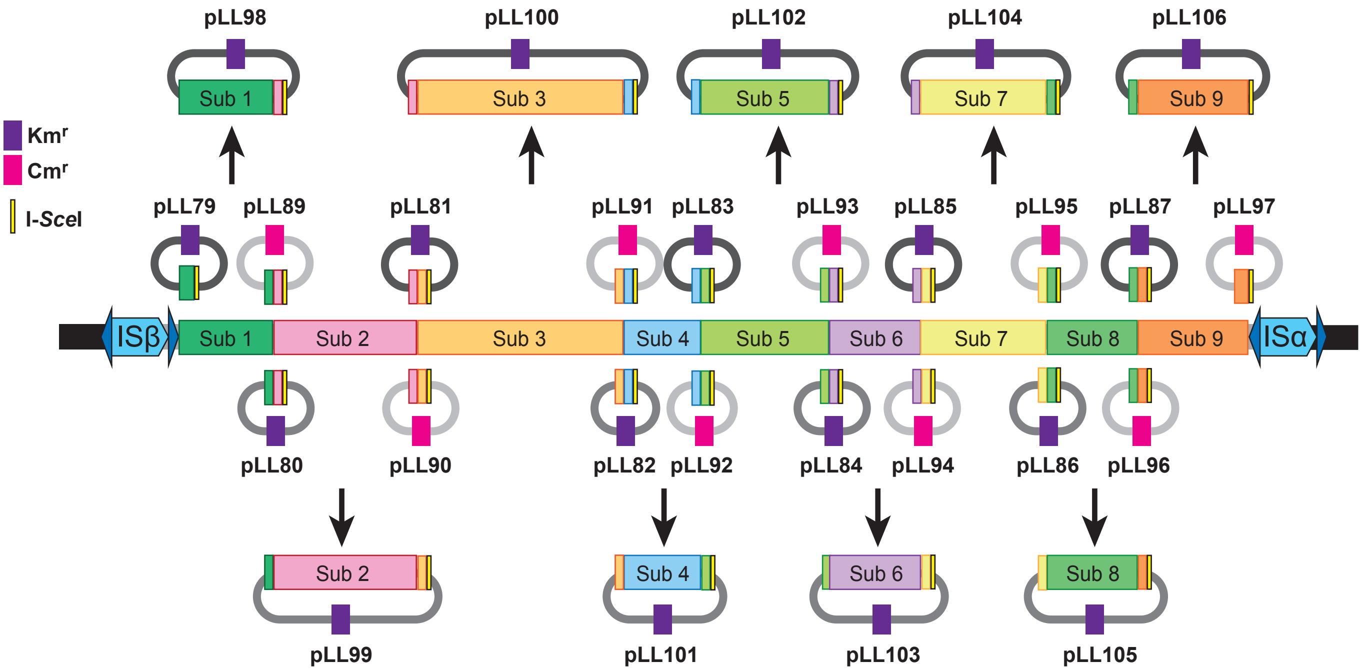

Supplemental Figure 1. Schematic of modified plasmid rescue strategy used to clone each subregion

The 208.6 kb region was divided into subregions as follows:

Subregion 1 contains BTH_I2743 – BTH_I2729
Subregion 2 contains BTH_I2728 – BTH_I2714
Subregion 3 contains BTH_I2713 – BTH_I2687
Subregion 4 contains BTH_I2686 – BTH_I2674
Subregion 5 contains BTH_I2673 – BTH_I2657
Subregion 6 contains BTH_I2656 – BTH_I2641
Subregion 7 contains BTH_I2640 – BTH_I2623
Subregion 8 contains BTH_I2622 – BTH_I2607
Subregion 9 contains BTH_I2606 – BTH_I2587

Each plasmid is a suicide plasmid for *Burkholderia* species and contains a ~500 bp fragment of DNA corresponding to a junction between subregions, as indicated by the colored boxes, and an I-*Sce*I restriction endonuclease site (yellow box outlined in black), as well as a gene encoding either kanamycin resistance (purple box) or chloramphenicol resistance (dark pink box). Plasmids containing subregions that were generated are shown across the top and bottom.

bioRxiv preprint doi: https://doi.org/10.1101/2024.12.09.627470; this version posted December 9, 2024. The copyright holder for this preprint (which was not certified by peer review) is the author/funder, who has granted bioRxiv a license to display the preprint in perpetuity. It is made available under aCC-BY 4.0 International license.

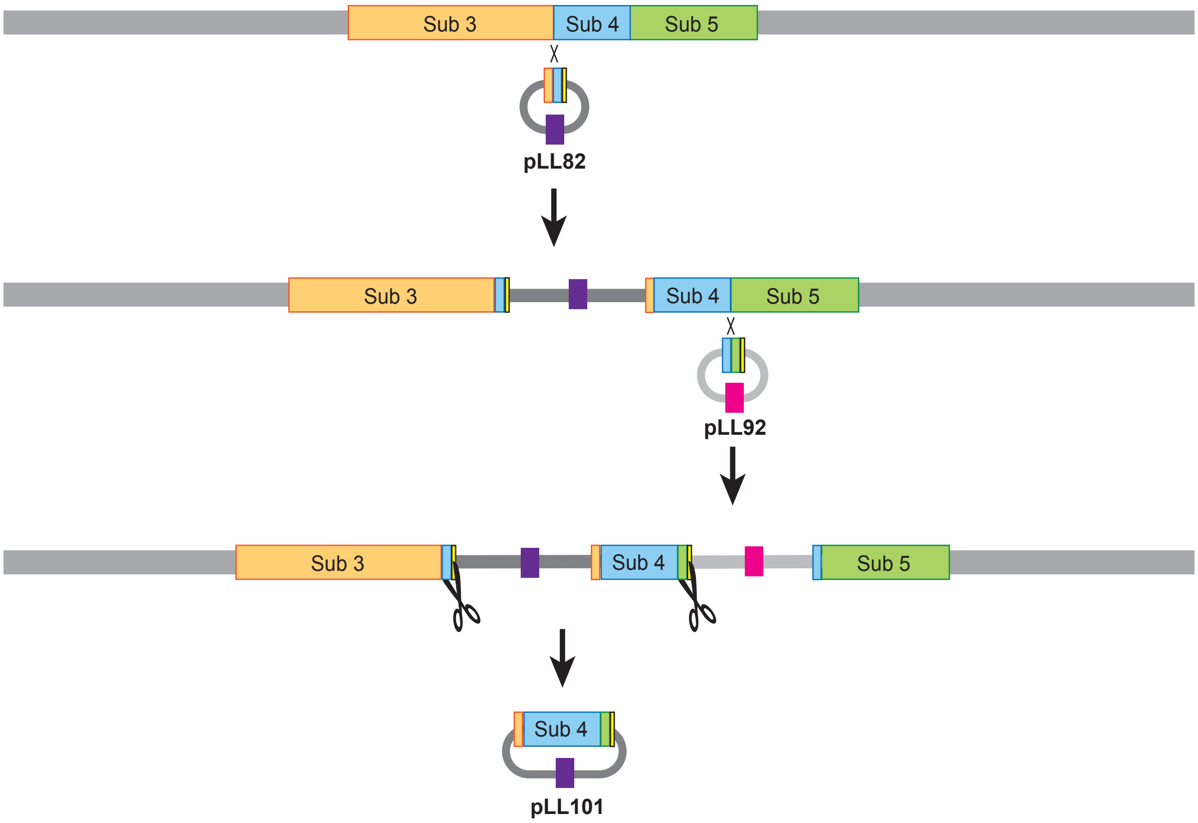

Supplemental Figure 2. Schematic of the modified plasmid rescue scheme

The plasmid rescue scheme to clone subregion 4 is shown. First, a Kmr cointegrant containing pLL82, which integrates at the junction between subregion 3 and subregion 4 was obtained. Then, a Cmr derivative of that strain containing pLL92 integrated at the junction between subregion 4 and subregion 5 was obtained. Genomic DNA was obtained from the strain containing both cointegrated plasmids, digested with I-*Sce*I and the fragmented DNA was ligated and used to transform E. coli DH5α. *E. coli* colonies were screened for those containing pLL101. All strains and plasmids were confirmed to be as expected by PCR and/or DNA sequence analysis.

bioRxiv preprint doi: https://doi.org/10.1101/2024.12.09.627470; this version posted December 9, 2024. The copyright holder for this preprint (which was not certified by peer review) is the author/funder, who has granted bioRxiv a license to display the preprint in perpetuity. It is made available under aCC-BY 4.0 International license.

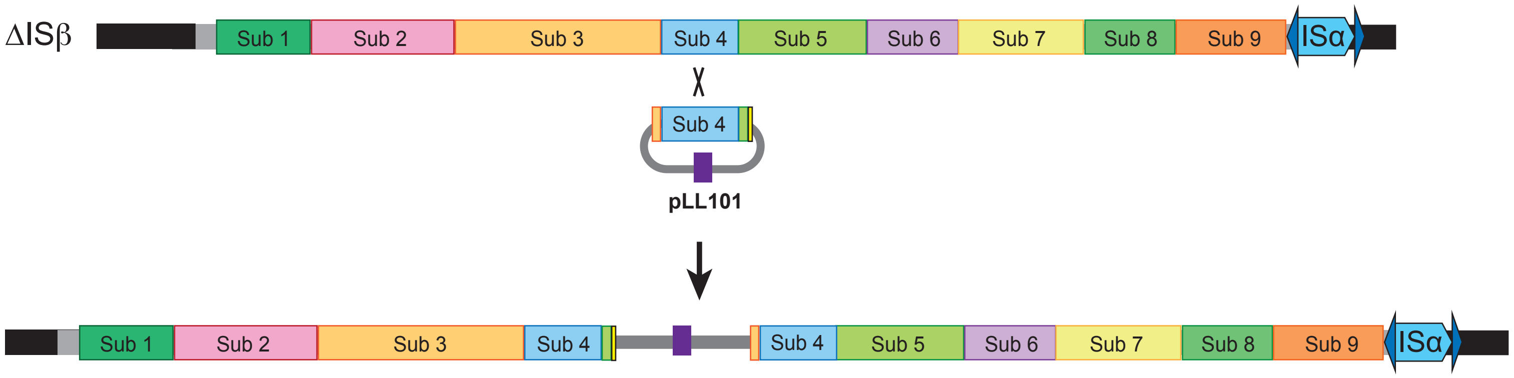

Supplemental Figure 3. Schematic of constructing strains containing a duplication of only a single subregion

A schematic of constructing a strain containing a duplication of subregion 4 is shown. Plasmid pLL101 was delivered to *Bt*E264 ΔISβ, which cannot duplicate the 208.6 kb region, by conjugation and Kmr cointegrants were obtained. Integration of pLL101 into subregion 4 was confirmed by PCR.
